# Prevalence of corneal arcus and associated factors in a German population—Results from the Gutenberg Health Study

**DOI:** 10.1371/journal.pone.0255893

**Published:** 2021-09-21

**Authors:** Joanna Wasielica-Poslednik, Ulrike Hampel, Lisa Ries, Ruah Faysal, Andreas Schulz, Jürgen H. Prochaska, Philipp S. Wild, Irene Schmidtmann, Thomas Münzel, Manfred E. Beutel, Karl J. Lackner, Norbert Pfeiffer, Alexander K. Schuster

**Affiliations:** 1 Department of Ophthalmology, University Medical Center of the Johannes Gutenberg- University Mainz, Mainz, Germany; 2 Preventive Cardiology and Preventive Medicine, Department of Cardiology, University Medical Center of the Johannes Gutenberg-University Mainz, Mainz, Germany; 3 Department of Cardiology–Cardiology I, University Medical Center Mainz, Mainz, Germany; 4 Center for Thrombosis and Hemostasis, University Medical Center of the Johannes Gutenberg-University Mainz, Mainz, Germany; 5 DZHK (German Center for Cardiovascular Research), Partner Site Rhine-Main, Mainz, Germany; 6 Institute for Medical Biostatistics, Epidemiology and Informatics, University Medical Center of the Johannes Gutenberg-University Mainz, Mainz, Germany; 7 Department of Psychosomatic Medicine and Psychotherapy, University Medical Center Mainz, Mainz, Germany; 8 Institute of Clinical Chemistry and Laboratory Medicine, University Medical Center Mainz, Mainz, Germany; University of Milano, ITALY

## Abstract

**Purpose:**

We aimed to determine the prevalence of corneal arcus and to identify associated factors in the general population of Germany.

**Methods:**

The Gutenberg Health Study (GHS) is a population-based cohort study in Germany, which includes an ophthalmological assessment. Refraction, distance-corrected visual acuity, non-contact tonometry and anterior segment imaging were performed for the five-year follow-up examination. Anterior segment photographs were graded for the presence of corneal arcus. Prevalence estimates were computed, and multivariable logistic regression analysis was applied to determine associated factors for corneal arcus including sex, age, spherical equivalent, central corneal thickness, intraocular pressure (IOP), socio-economic status, smoking, BMI, systolic and diastolic arterial blood pressure, HbA1c, HDL-C, LDL-C, triglyceride, and lipid modifying agents.

**Results:**

A total of 9,850 right and 9,745 left eyes of 9,858 subjects (59.2±10.8 years), 49.0% females were included in this cross-sectional analysis. 21.1% of men (95%-CI: 20.0%– 22.3%) had a corneal arcus in at least one eye, and 16.9% (95%-CI: 15.9%– 18.0%) of women. In multivariable analyses, the presence of corneal arcus was associated with male gender (OR = 0.54 for female, p<0.0001), higher age (OR = 2.54 per decade, p<0.0001), smoking (OR = 1.59, p<0.0001), hyperopia (OR = 1.05 per diopter, p<0.0001), thinner cornea (OR = 0.994 per μm, p<0.0001), higher IOP (OR = 1.02, p = 0.039), higher HDL-C-level (OR = 2.13, p<0.0001), higher LDL-C-level (OR = 1.21, p<0.0001), and intake of lipid modifying agents (OR = 1.26, p = 0.0001). Arcus was not associated with socio-economic status, BMI, arterial blood pressure, and HbA1c.

**Conclusions:**

Corneal arcus is a frequent alteration of the cornea in Germany and is associated with ocular parameters and systemic parameters of dyslipidemia.

## Introduction

**Fig 1:**
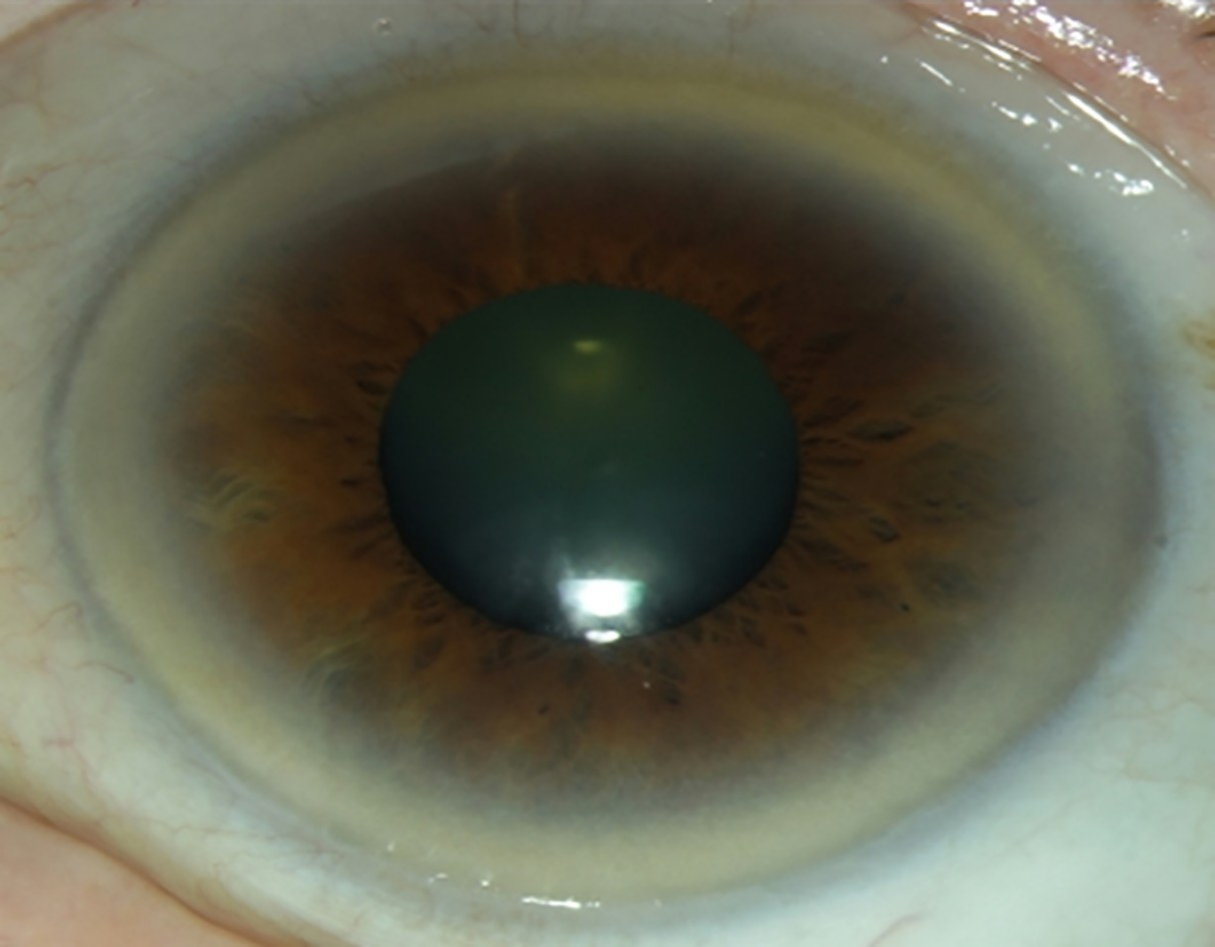
Example of a corneal arcus.

Corneal arcus is a common ophthalmological finding, which may be associated with dyslipidemia or may occur independently with normal ageing processes. This mostly bilateral grey-white-yellowish opacity appears in the corneal periphery, separated from the limbus by a clear corneal zone (lucid interval of Vogt) with a sharp edge on its limbal margin and a less well-defined edge on its central margin (Fig 1) [1]. The pathophysiology of arcus stems from increased permeability of vessels of the conjunctiva and episclera which are adjacent to the anterior cornea and the ciliary vessels adjacent to the posterior cornea. These are involved in transport, delivery and removal of lipids and lipoproteins from the corneal tissue [2]. Permeability of the vascular tissue to circulating lipids may increase due to vascular pathology, limbal vascular anomalies, perilimbal inflammation, increased temperature, or a tumor. Hence, lipids are initially deposited in the warmest parts of the cornea, which are the superior and inferior periphery. Hyperlipoproteinemia is the most common systemic risk factor predisposing to this ocular lipid deposition.

In humans, the lipids are accumulated almost exclusively in the extracellular space. The initial arcs may progress circumferentially. The earliest site of the lipid deposition in eyes with visible arcus is found in the deeper layers of corneal stroma and sclera and later on in the superficial cornea adjacent to the limbal vascular plexus [3]. In advanced cases, the entire thickness of the cornea including both Bowman and Descemet membranes may show lipid deposition. There are some early reports regarding cholesterol crystals within the anterior chamber [4].

The corneal stroma resembles an artery wall in its structure, consisting of dense connective tissue as well as keratocytes and smooth muscle cells [5]. Disruption of local cholesterol homeostasis leads to extracellular accumulation of cholesterol in the corneal tissue and, as well as atherosclerosis [6], with increased low-density lipoprotein cholesterol (LDL-C) levels underpinning these processes. LDL-C is a major source of cholesteryl ester and apolipoprotein (Apo) B that are found in arcus [7]. The degree of arcus correlates positively with hypercholesterolemia caused by an excess of LDL-C [8]. Premature arcus may also be observed by primary or secondary hypertriglyceridemia caused by high levels of very low-density lipoproteins [2].

Previous studies have demonstrated the presence of corneal arcus to be associated with age [9, 10], male sex [11], black race [12], genotype [13], elevated plasma LDL-C level [14], alcohol intake [15], diabetes mellitus [7], smoking [16], blood pressure [8], BMI [14], lower HDL-C level [17] and deficiency of Apo A-1 [18]. Other studies, however, did not find correlations between arcus and obesity, hematocrit, serum triglyceride, HDL-C- or VLDL-levels [6]. Appearance of arcus before the age of 50 most notably in hyperlipidemic men correlates with premature development of cardiovascular diseases [19]. It is unclear whether arcus represents a risk factor of cardiovascular diseases independent of hyperlipidemia in people over 50 years of age [20].

Little is known about correlations between arcus and other ocular parameters. In this population-based study, we determine the prevalence of corneal arcus in our cohort and analyze its relation to the above- mentioned systemic risk factors. In addition, correlations between corneal arcus and ocular parameters such as corneal thickness, refractive error, and intraocular pressure (IOP) are evaluated.

## Materials and methods

The Gutenberg Health Study (GHS) is a prospective, population-based, observational cohort study conducted in the Rhine-Main region in Midwestern Germany. A study sample of 15,010 participants was drawn in waves of equal stratification to meet a standardized recruiting. More details of the study design are described by Höhn et al. [21]. For each participant, a comprehensive ophthalmological work-up was conducted. Non-cycloplegic refraction (Humphrey Automated Refractor/Keratometer (HARK) 599, Carl Zeiss Meditec AG, Jena, Germany) was measured and spherical equivalent was computed. Central corneal thickness was measured as part of ocular biometry with Lenstar LS900 (Haag-Streit Diagnostics, Koeniz, Switzerland). Non-contact tonometry (Nidek NT-2000, Nidek Co, Japan) was performed and repeated three times. Anterior segment photography was performed of both eyes at the 5-year follow-up examination. General anthropometric parameters including body height and body weight were determined and smoking habits were recorded. All examinations were performed by experienced study nurses in accordance with standardized operation procedures.

The study protocol and study documents were approved by the local ethics committee of the Medical Chamber of Rhineland-Palatinate, Germany (reference no. 837.020.07; original vote: 22.3.2007, latest update: 20.10.2015). According to the tenets of the Declaration of Helsinki, written informed consent was obtained from all participants prior to entering the study.

The GHS is a joint project of internal medicine, ophthalmology, psychosomatic medicine and epidemiology at the Johannes Gutenberg-University Mainz, Germany.

The written informed consent of GHS study participants does not approve public access to the data. This was requested by the local data protection officer and ethics committee (local ethics committee of the Medical Chamber of Rhineland-Palatinate, Germany). Access to data is made in accordance with the ethics vote is offered upon request at any time. Interested researchers can make their requests to the Principal Investigators of the Gutenberg Health Study (email: info@ghs-mainz.de).

### Study sample

This study sample was recruited from the five-year follow-up of the GHS cohort including subjects with an age range from 40 to 80 years at time of examination. According to this criterion, 12.423 (83%) subjects came for this follow-up examination and were eligible for this study.

### Data and statistical analysis

Anterior segment images were examined for the presence of corneal arcus by two trained graders (LR, RF) at Mainz Ophthalmic Reading Center. In addition, the extent of arcus was graded as corneal involvement of <180°, ≥ 180° or ≥180° with dense involvement. Masked intra- and inter-rater comparison were performed. Kappa-statistics were calculated to evaluate reliability. All questionable findings were evaluated by the supervisor (AKS).

Absolute and relative frequencies were computed for categorical variables. Median, inter quantile range, minimum and maximum were calculated for all continuous variables. For variables found to be within a normal distribution, mean and standard deviation were computed. Non-responder analysis was carried out to compare systemic and ocular characteristics between subjects with and without gradable anterior segment photographs. Diabetes was defined as having a respective diagnosis and treatment by a physician or if individuals showed HbA1c-level ≥6.5% [47.5 mmol/mol].

First, prevalence of corneal arcus was determined for the presence in right eyes, left eyes, as well as for unilateral versus bilateral cases. The weighted prevalence for the German population was computed based on the German population distribution from 2015 (December 31^st^).

Associated factors were evaluated using a generalized estimating equation model with consideration of the correlation structure between both eyes of the subjects.

A two-step analysis was performed. In the first model, we examined associations to anthropometric and ocular parameters and cardiovascular risk factors. This model included sex, age, spherical equivalent, central corneal thickness, socio-economic status, smoking, BMI, systolic and diastolic arterial blood pressure, HbA1c-level, HDL-C-level, LDL-C-level, triglyceride-level and lipid lowering medication. Lipid modifying agents, coded as c10 according to the anatomical therapeutic chemical classification system (ATC code), included HMG CoA reductase inhibitors, fibrates, bile acid sequestrates, nicotinic acid and derivatives, as well as other lipid modifying agents.

The second model included only those subjects who had a corneal arcus. This model evaluated, whether there are differences between those with a dense corneal arcus comparing to those with a slightly pronounced corneal arcus.

All p-values should be regarded as a continuous parameter that reflects the level of evidence and are therefore reported exactly. Data were processed by statistical analysis software (R version 3.1.1 [2014-07-10]).

## Results

In this cross-sectional study, 9,850 right and 9,745 left eyes of 9,858 subjects (49.0% female) were included. The mean age of the study participants was 59.2±10.8 years (range 40–80 years). The study sample is further characterized in Table 1. Non-responder analysis revealed that the included subjects were slightly younger, while other factors showed a comparable distribution (S1 Table).

**Table 1:** Characteristics of the analysis sample. Data from the population-based Gutenberg Health Study (2012–2017).

Variable	All	Men	Women
**n**	9858	5029	4829
**Age [y]**	59.2±10.8	59.4±10.8	58.9±10.7
**Socio-economic status**	13.12±4.43	13.79±4.45	12.43±4.30
**BMI [kg/m^2^]**	27.5±5.0	28.0±4.4	27.0±5.5
**Mean arterial blood pressure [mmHg]**	97.3±10.7	99.0±10.2	95.6±10.9
**Cardiovascular risk factors**			
**Obesity (yes)**	25.7% (2530)	26.7% (1342)	24.6% (1188)
**Diabetes (yes)**	10.0% (983)	12.4% (624)	7.5% (359)
**Smoking (yes)**	15.1% (1485)	15.8% (794)	14.3% (691)
**Hypertension (yes)**	53.3% (5244)	58.5% (2932)	47.9% (2312)
**Dyslipidemia (yes)**	43.6% (4286)	52.8% (2655)	33.9% (1631)
**Family history of myocardial infarction/Stroke (yes)**	23.4% (2306)	21.7% (1091)	25.2% (1215)
**Lab parameters:**			
**HbA1c [%]**	5.60 (5.30/5.80)	5.60 (5.30/5.90)	5.50 (5.30/5.80)
**Cholesterol [mmol/l]**	222.1±42.4	214.2±41.3	230.4±42.0
**HDL-C [mmol/l]**	1.52 +/- 0.41	1.34 +/- 0.33	1.71 +/- 0.40
**LDL-C [mmol/l]**	3.62 +/- 0.95	3.54 +/- 0.94	3.71 +/- 0.96
**Triglycerides [mmol/l]**	1.16 (0.86/1.58)	1.26 (0.93/1.77)	1.07 (0.81/1.43)
**Ophthalmological characteristics**			
**DCVA [logMAR] OD**	0.10 (0/0.22)	0.10 (0/0.10)	0.10 (0/0.22)
**DCVA [logMAR] OS**	0 (0/0.10)	0 (0/0.10)	0.10 (0/0.22)
**IOP [mmHg] OD**	14.74±2.96	14.85±3.08	14.62±2.84
**IOP [mmHg] OS**	14.83±3.00	14.96±3.11	14.69±2.87
**Spherical equivalent [dpt] OD**	-0.12 (-1.25/0.88)	-0.12 (-1.25/0.88)	-0.12 (-1.25/0.88)
**Spherical equivalent [dpt] OS**	-0.12 (-1.25/0.88)	-0.12 (-1.25/0.88)	-0.12 (-1.25/0.88)
**Central corneal thickness [μm] OD**	552±36	553±35	550±36

**Fig 2:**
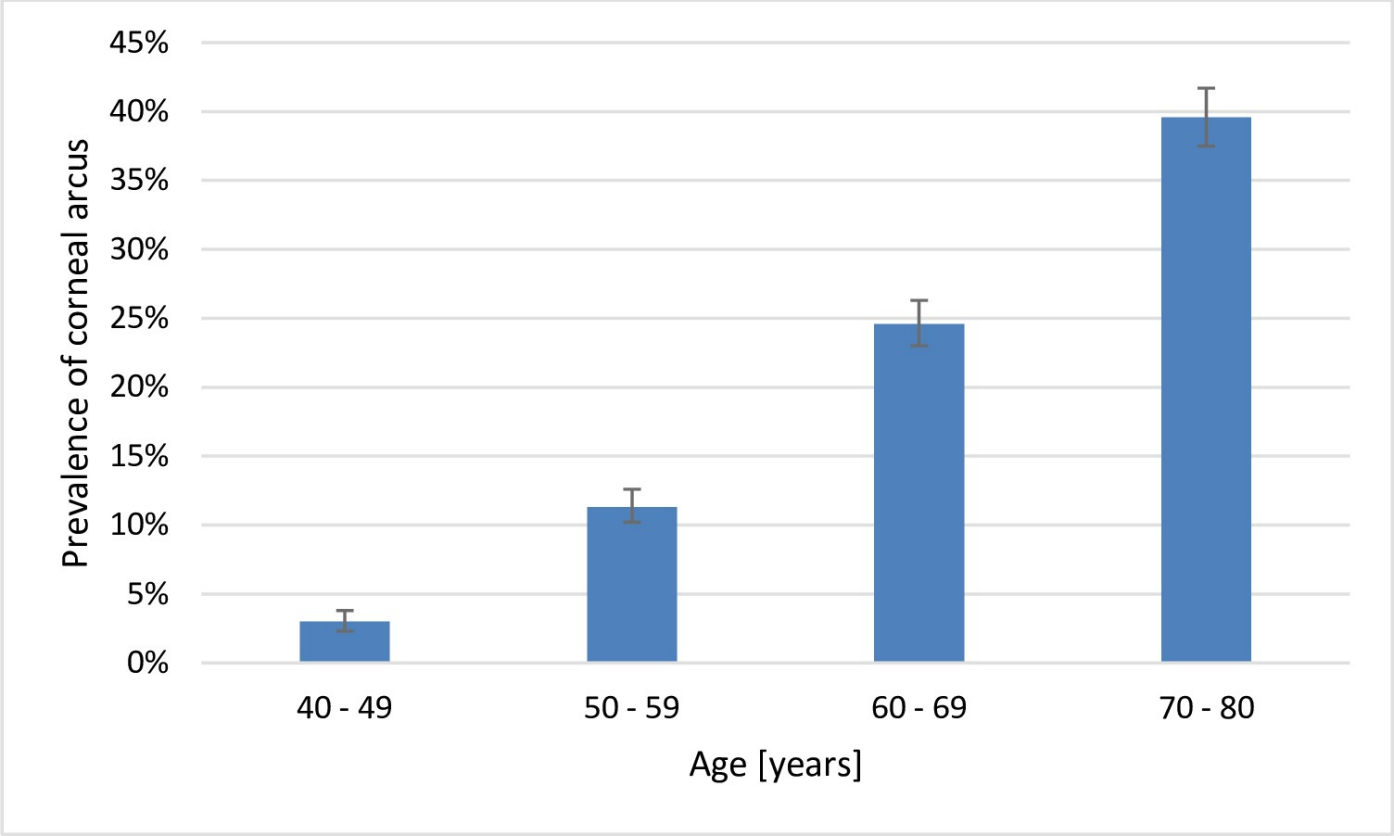
Prevalence of corneal arcus. Data from the population-based Gutenberg Health Study (2012–2017; n = 9.958). Prevalence estimates including 95% confidence interval.

1879 participants (19.1%; 95%-CI: 18.3–19.9%) had corneal arcus in at least one eye, the weighted prevalence for Germany at age 40 to 80 years was 17.4% (95%-CI: 16.6–18.1%). The prevalence increased with age (Fig 2). Of those individuals with corneal arcus, 66.9% of right eyes had corneal arcus with <180°, 18.8% had corneal arcus ≥180° and 14.4% had corneal arcus ≥180° with a dense characteristic. Left eyes with arcus demonstrated 68.6% <180°, 17.2% ≥180° and 14.2% ≥180° with a dense characteristic.

In multivariable analyses, the presence of corneal arcus was associated with male gender (OR = 0.54 for women, p<0.0001), higher age (OR = 2.54 per decade, p<0.0001), smoking (OR = 1.59, p<0.0001), hyperopia (OR = 1.05 per diopter, p<0.0001), thinner cornea (OR = 0.994 per μm, p<0.0001), higher IOP (OR = 1.02, p = 0.034), higher HDL-C-level (OR = 2.13, p<0.0001), higher LDL-C-level (OR = 1.21, p<0.0001), and intake of lipid modifying agents (OR = 1.26, p = 0.0001). Arcus was not associated with socio-economic status, BMI, arterial blood pressure, and HbA1c (Table 2).

**Table 2:** Investigation of associations between corneal arcus and cardiovascular parameters. Data from the population-based Gutenberg Health Study (2012–2017). Multivariable logistic regression analysis with Generalized Estimation Equations (GEE) modelling was conducted to determine associated factors.

N = 18,484	OR	95%-CI	p-value
Sex (Women)	0.54	0.49–0.60	**<0.0001**
Age [10y]	2.54	2.40–2.68	**<0.0001**
Socioeconomic status	1.00	0.990–1.01	0.93
Smoking	1.59	1.40–1.80	**<0.0001**
Body-Mass Index [kg/m^2^]	0.994	0.984–1.00	0.30
Systolic blood pressure [mmHg]	0.997	0.993–1.00	0.086
Diastolic blood pressure [mmHg]	1.01	0.999	0.12
HbA1c [%]	1.01	0.946–1.07	0.80
HDL-C [mmol/l]	2.13	1.87–2.43	**<0.0001**
LDL-C [mmol/l]	1.21	1.15–1.27	**<0.0001**
Triglycerides (log[mmol/l])	1.12	0.99–1.27	0.063
Lipid modifying agents (c10)	1.26	1.12–1.41	**0.00010**
Intraocular pressure [mmHg]	1.02	1.00–1.03	**0.034**
Spherical equivalent [dpt]	1.05	1.03–1.07	**<0.0001**
Central corneal thickness [μm]	0.994	0.992–0.995	**<0.0001**

When comparing subjects with dense and not-dense corneal arcus, those with dense corneal arcus were more likely male (OR = 0.54 for women, p<0.0001), older (OR = 1.72 per decade, p<0.0001), more hyperopic (OR = 1.11 per diopter, p<0.0001), had a thinner central cornea (OR = 0.99 per μm, p<0.0001), were more likely smokers (OR = 1.46, p = 0.02), had lower socio-economic status (OR = 0.97, p = 0.046) and a higher HDL-C-level (OR = 1.49, p = 0.008) (Table 3).

**Table 3:** Investigation of associations with dense corneal arcus. Data from the population-based Gutenberg Health Study (2012–2017). Multivariable logistic regression analysis with GEE modelling was conducted to determine associated factors. As control group, those with corneal arcus without dense characteristic were included.

N = 3,047	OR	95%-CI	p-value
Sex (Women)	0.535	0.421–0.680	**<0.0001**
Age [10y]	1.72	1.47–2.02	**<0.0001**
Socioeconomic status	0.974	0.950–0.999	**0.046**
Smoking	1.46	1.06–2.01	**0.020**
Body-Mass Index [kg/m^2^]	1.02	0.987–1.05	0.29
Systolic blood pressure [mmHg]	1.00	0.995–1.01	0.52
Diastolic blood pressure [mmHg]	0.995	0.981–1.01	0.50
HbA1c [%]	0.853	0.693–1.05	0.13
HDL-C [mmol/l]	1.49	1.11–2.01	**0.0084**
LDL-C [mmol/l]	1.09	0.965–1.23	0.17
Triglycerides (log[mmol/l])	0.985	0.728–1.33	0.92
Lipid modifying agents (c10)	0.919	0.702–1.20	0.54
Intraocular pressure [mmHg]	0.999	0.960–1.04	0.95
Spherical equivalent [dpt]	1.11	1.05–1.16	**<0.0001**
Central corneal thickness [μm]	0.990	0.987–0.994	**<0.0001**

Our study included a subpopulation of 983 diabetic persons (40 subjects with type 1 diabetes, 728 of type 2 diabetes, one with diabetes after pancreatitis, 20 with unknown type of diabetes and 194 with screening-detected diabetes).

## Discussion

From the GHS cohort, the calculated prevalence of corneal arcus in the German population aged 40 to 80 was 17.4%. These findings are comparable to results of a study from an Iranian population showing a prevalence of 23.3% [22]. In a younger Indian population, the prevalence was reported to be 10.7% [23], while in the Singapore Malay Eye Study the prevalence was 57.9%, showing a ethnic differences [24]. In the Copenhagen City Heart Study–a mainly Caucasian study investigating almost 13,000 Danish people in the late seventies–the overall prevalence of corneal arcus at baseline was 24.8% and increased with age to 80% over the age of 70. It was lower in women than in men (20.1% vs. 30.2%) [25]. Prevalence of circumferential corneal arcus in an Australian older population (>49 years) in the Blue Mountains Eye Study was 64.8% [26]. In 1965, Rifkind et al. found the prevalence of corneal arcus in a male population to be as high as 75% at age 60 to 70 [8]. Similar to our study, the prevalence of corneal arcus was lower in females. The high prevalence of corneal arcus observed in the last century may be lowered nowadays by lipid-lowering medication, which has gained popularity for the treatment of dyslipidemia and reduced the risk of cardiovascular diseases remarkably in the last three decades [27]. Similarly, we found an association between the intake of these lipid-lowering agents and the presence of corneal arcus. Thus, arcus most likely originates from a dyslipidemia and does not seem to regress despite lipid-lowering medication.

There are only a few studies on the co-existence of corneal arcus with other ocular parameters. In our analysis, the presence of corneal arcus was associated with a thinner central cornea. Similar results are reported in the Singapore Malay Eye Study [28]. This may partly be explained by systemic factors, such as age, sex, body mass index, diabetes, chronic kidney disease or metabolic syndrome which are also associated with central corneal thickness [29, 30]. Nevertheless, our multivariable analysis did show central corneal thickness as an independently associated factor.

We speculate that accumulation of lipids, which begins in the deeper corneal layers, reaches the superficial and hence visible corneal layers more readily in thinner corneas, manifesting in the demonstratable presence of arcus.

There is no widely accepted explanation for the association of corneal arcus with higher IOP. Similar to our results, the Singapore Malay Eye Study showed a higher IOP in eyes with corneal arcus [29]. This might be due to alterations in biomechanical properties such as corneal resistance factor and corneal hysteresis, which are reported to be related to aging and other corneal opacities [31, 32]. Such alterations may lead to a stiffening of the cornea and may influence the IOP measurement [33]. Ayhan et al. reported altered corneal biomechanical characteristics in eyes with corneal arcus [34]. As lower central corneal thickness and higher IOP are both risk factors for conversion of ocular hypertension into glaucoma, further studies should evaluate whether this risk is modified by the presence of corneal arcus [35].

In our study, corneal arcus correlated significantly with dyslipidemia parameters such as higher LDL-C-levels. Surprisingly, arcus also correlated with higher HDL-C-levels. Similar to our findings, in the Copenhagen City Heart Study plasma concentrations of total cholesterol, LDL-C, triglycerides and lipoprotein (a) were higher in people with corneal arcus. HDL-C and Apolipoprotein A1 levels did not differ between people with and without arcus. In the Blue Mountains Eye Study corneal arcus correlated with total cholesterol, hypertriglyceridemia, but not with HDL-C level. We speculate that the positive correlation between corneal arcus and increased HDL-C-level may result from an appropriate adjustment of the lipid-lowering therapy and normalization of the HDL-C-level in our study population. Development of corneal arcus probably proceeds the diagnosis of hyperlipoproteinemia and initiation of lipid-lowering therapy.

One limitation of our study is that we were not able to differentiate corneal arcus from rare entities such as genetic diseases affecting lipid clearing mechanisms, hematological disorders or embryotoxon by osteogenesis imperfecta. Genetic mutations in *ABCA1* (Tangier disease), *LCAT* (familial lecithin:cholesterol acyltransferase deficiency), *ApoA1* (familial apolipoprotein (Apo) A1 deficiency), and *UBIAD1* (Schnyder corneal dystrophy, SCD) all result in corneal accumulation of lipids including cholesterol. SCD characterized by a crystalline or non-crystalline central haze, which progresses to corneal arcus in the third decade and to the midperipheral haze in the late fourth decade, results from increased deposition of cholesterol and phospholipids in the cornea (SCD) [36]. Monoclonal gammopathies may lead to accumulation of immunoglobulins or of free light chains in form of a corneal arcus as well [37]. Nevertheless, these diseases are very rare and their influence on our estimates is rather low due to the population-based study design. Corneal arcus was graded on anterior segment photographs on which illumination, contrast and field of view may have had an influence. We therefore performed several sensitivity analyses and found similar results. Amini and Ameri proposed a deep learning based automatic recognition of corneal arcus [38]. Their model achieved a high accuracy in classifying images with and without arcus. Such methods may be considered in future population-based studies. Furthermore, our study investigates a mainly Caucasian population in Germany at age 40 to 80 years, and therefore these findings may not apply to other ethnicities or ages outside this range.

To conclude, our study confirmed the relationship between corneal arcus and dyslipidemia. The weighted prevalence of corneal arcus in the German population is 17.4% and seems to be lower than in previous studies, which may be due to broad application of lipid-lowering medication.

## Supporting information

S1 Table Item-non-responder analysis. Data from the population-based Gutenberg Health Study (2012–2017).
(PDF)
