# Supplementary material for: Prevalence of corneal arcus and associated factors in a German population—Results from the Gutenberg Health Study
Source: PLoS One. 2021 Sep 21;16(9):e0255893. doi: 10.1371/journal.pone.0255893 (PMC8454945; doi:10.1371/journal.pone.0255893)
Supplement: S1 Table — Data from the population-based Gutenberg Health Study (2012–2017). (PDF) [file pone.0255893.s001.pdf]

	**Subjects with corneal arcus grading** (9927)	**Subjects without corneal arcus grading** (2496)
Anthropometric parameters		
Sex (women)	49.0%	48.3%
Age	59.2±10.8	60.6±11.1
Blood parameters		
HbA_{1c} [%]	5.60 (5.30/5.80)	5.60 (5.30/5.90)
HDL [mg/dl]	58.6±15.8	58.7±15.5
LDL [mg/dl]	139.4±36.6	138.7±36.4
Trigylzeride [mg/dl]	105 (78/144)	106 (79/148)
Socioeconomic parameters		
Socioeconomical status	13.12±4.43	12.66±4.45
Smoking (yes)	15.1%	15.3%
Cardiovascular risk factors		
Obesity (yes)	25.7%	27.3%
Diabetes (yes)	10.0%	12.8%
Arterial hypertension (yes)	53.3%	56.2%
Dyslipidemia (yes)	43.6%	46.8%
